# Multimodal bioimaging using nanodiamond and gold hybrid nanoparticles

**DOI:** 10.1038/s41598-022-09317-3

**Published:** 2022-03-29

**Authors:** Yu-Chung Lin, Elena Perevedentseva, Zhe-Rui Lin, Chia-Chi Chang, Hsiang-Hsin Chen, Shun-Min Yang, Ming-Der Lin, Artashes Karmenyan, Giorgio Speranza, Luca Minati, Christoph Nebel, Chia-Liang Cheng

**Affiliations:** 1 Department of Physics, National Dong Hwa University, Hualien, Taiwan; 2 Institute of Physics, Academia Sinica, Taipei, Taiwan; 3 P. N. Lebedev Physics Institute, Russian Academy of Sciences, Moscow, Russia; 4 Department of Molecular Biology and Human Genetics, Tzu-Chi University, Hualien, Taiwan; 5 Bruno Kessler Institute (FBK), Trento, Italy; 6 Department of Materials Engineering and Industrial Technologies, University of Trento, Trento, Italy; 7 Institute for Photonics and Nanotechnologies of National Research Council (CNR-IFN), CSMFO Lab, Trento, Italy; 8 IMMAGINA BioTechnology S.R.L, Via Sommarive 18, Povo, Trento, Italy; 9 Diamond and Carbon Applications GmbH (DiaCarA), Freiburg, Germany

**Keywords:** Biophysics, Imaging, Fluorescence imaging

## Abstract

Hybrid core–shell nanodiamond-gold nanoparticles were synthesized and characterized as a novel multifunctional material with tunable and tailored properties for multifunctional biomedical applications. The combination of nanostructured gold and nanodiamond properties afford new options for optical labeling, imaging, sensing, and drug delivery, as well as targeted treatment. ND@Au core–shell nanoparticles composed of nanodiamond (ND) core doped with Si vacancies (SiV) and Au shell were synthesized and characterized in terms of their biomedical applications. Several bioimaging modalities based on the combination of optical and spectroscopic properties of the hybrid nano-systems are demonstrated in cellular and developing zebrafish larvae models. The ND@Au nanoparticles exhibit isolated ND’s Raman signal of sp^3^ bonded carbon, one-photon fluorescence of SiV with strong zero-phonon line at 740 nm, two-photon excited fluorescence of nanogold with short fluorescence lifetime and strong absorption of X-ray irradiation render them possible imaging agent for Raman mapping, Fluorescence imaging, two-photon Fluorescence Lifetime Imaging (TP-FLIM) and high-resolution hard-X-ray microscopy in biosystems. Potential combination of the imaging facilities with other theranostic functionalities is discussed.

## Introduction

Multifunctionality is one of the advantages of hybrid nanoparticle (NP) used in biomedical studies. Nanoparticles with multimodal imaging and sensing capacity can combine drug delivery capabilities to other medical treatments for theranostic applications. The possibility to integrate several functionalities for one nanoparticle-based complex provides promising potential in a wide range of applications. One approach is to design the NP with pre-established necessary properties, such as a hybrid structure for a combination of complementary facilities of the involved components and obtaining synergistic properties. Among such complex nano-systems, rapid and diverse evolution takes place in the development of hybrid NP with Au component^1^. Combining Au with porous silica^2^, silicon^3^, iron oxides^4^, polymers^5^ or other organic macromolecules, e.g. transition metals complexes^6^, different kinds of carbon nanomaterials^7–10^ etc., have been described previously.

Among other currently used NP, hybrid gold-nanodiamond are novel multifunctional materials with tunable and tailored properties that have been considered for various biomedical applications. By combining nano-gold and nanodiamond, the synergetic properties of both materials can provide new options to combine optical labeling, imaging, sensing, and drug delivery as well as targeted treatment^10^. Both nanoscale gold and diamond materials have been demonstrated to be promising materials for use in biomedical researches and already in some medical applications. Additionally, both of them are considered in general biocompatible, and their in-vitro and in-vivo toxicity were thoroughly studied recently^11–15^.

Gold nanoparticles (AuNP) for biomedical applications are designed in a very wide range of sizes, shapes, and properties^16–18^. The AuNP surface can be conjugated with drugs and other molecules for treatment, targeted delivery and specific interaction^16,17^. Due to the plasmonic properties, AuNP can be designed as imaging agents with different methods of detection such as photoacoustic, enhanced scattering, two-photon excited luminescence, improving the contrast in transmission electron microscopy (TEM) images^17–20^, and for biosensing etc.^16,17^. Colorimetric sensing, Foster Resonance Energy Transfer (FRET)-based detection, electrical and electrochemical sensing^17^ also have been demonstrated for different Au nanoparticles. The applications for photothermal and photodynamic therapy have also been attempted^16,21^.

Nanodiamond (ND) also has been studied and considered potentially a very promising material for biomedical applications due to their physical and chemical properties and variability in their size and structure^11,12,22^. The surface properties of NDs allow the functionalization of the particles and conjugation with molecules of interests for drug delivery and to interact with biotargets^22,23^. The optical and spectroscopic properties of NDs allow them to be used as imaging agent for different methods of bioimaging, based on NDs’ fluorescence^11,12,22^ and other properties, for example Raman scattering^12^. With applicability of ND for multimodal imaging and delivery monitoring, it has been used for sensing^24^, based on the electronic spin state in (NV)^−^ center that can be optically detected and used for sensitive nanoscale magnetometry. The main origin of ND fluorescence is color centers in the diamond core or the surface of ND^11,22,24,25^. The most studied and used for bioimaging and biosensing are nitrogen vacancy centers^22,25^ of both negatively charged (NV)^−^ and neutral (NV)^0^. Using high energy treatment, the number of the color centers can be increased and hence the fluorescence is enhanced^25^. In addition to (NV) centers, silicon vacancy center (SiV) also attracts attention recently due to emission in the near-infrared range with intense and narrow fluorescence zero phonon line (ZPL) at 740 nm^26,27^. Additionally, diamond intense sp^3^ carbons Raman peak can be exploited for Raman mapping that provides other imaging information^12^.

Hybrid materials can amplify the complementary components’ properties. The advantages of the synergy between gold and nanodiamond particles can be expected. However, the optical properties of the coupled Au and ND nanoparticles are not completely clear and predictable yet, leading to some contradictory results^10^. The ND-Au hybrid possess the plasmonic and photoacoustic properties of gold component^28–30^. In the presence of the nanodiamond the photoacoustic signal of the gold is amplified. The energy or charge transfer between energy-absorbing ND and nano-Au has been discussed. Additionally, it was found that combining Au with ND prevents Au nanostructure from degradation during photoacoustic imaging^28^. The same was observed with transmission electron microscopy (TEM) imaging^10^. Another synergy effect, the catalytic and sensor facilities are based on charge transfer process found only in the hybrid system^10^. Thus, Au-decorated NDs demonstrated tunable optical properties and local plasmonic resonance and were applied as a substrate for Surface Enhanced Raman Spectroscopy (SERS) with improved SERS effect compared to nano-Au alone^31^. For temperature measurements in nanoscale combining diamond and gold with a high localizing of thermal effect has been shown^29,32,33^. In the system of a single AuNP conjugated with a single ND, highly localized heating is realized, which allows the temperature detection combined with controlled plasmon-based photothermal therapy^34^. Synergetic effect of catalytical activity is observed in hybrid AuNP-NDs^35,36^. This effect is related to the charge transfer from gold nanoparticles to sp^2^ dangling bonds on ND surface^36^.

ND’s hybridization with metal have been found affecting the fluorescence^30,37^ and photoacoustic^33^ properties of ND. These effects can be used for ND-based hybrid’s fluorescence tuning for imaging and sensing. New facilities of different photonic and optoelectronic applications can be provided by the non-linear optical response of the ND-gold nanohybrids, that is significantly larger than that of pure ND and depends on the Au loading at picosecond visible light excitation^38^. The hybrid gold-nanodiamond are synthesized mostly in 2 forms, core–shell nanoparticles with ND core and Au shell^9,32,39^ and nanocomposites of ND and Au nanoparticles conjugated with covalent or non-covalent bonding^28,30,36,37^, sometimes referred to ND-decorated AuNP or gold-decorated ND^10^. Such hybrids can be also conjugated with additional NP component to increase functionalities^40^.

In this work we synthesized and characterized the functionalities of diamond-based nanostructures formed by SiV-doped ND cores coated with a gold shell (ND@Au). The samples were presented and characterized previously^39^. This NP hybrid combines abilities and advantages of fluorescent ND and of the nanostructured metal with plasmonic properties. We discuss the multifunctional bio application of ND@Au NP focusing on their applications for multimodal imaging based on the optical properties of both ND and gold.

## Results and discussion

### ND@Au characterization

SiV-doped nanodiamonds (ND) were synthesized using plasma enhanced chemical vapor deposition (PECVD) technique with a Si-wafer as Si-doping source. Au shell was synthesized via a gold reduction in the HAuCl_4_ water solution with reducing agent hydroxylamine hydrochloride NH_2_OH*HCl and ND as the seeds. The core–shell nanoparticles were synthesized using nanodiamond (ND) core enriched with Si vacancies (SiV) and Au shell (ND@Au) were characterized in terms of their biomedical applications.

**Figure 1:**
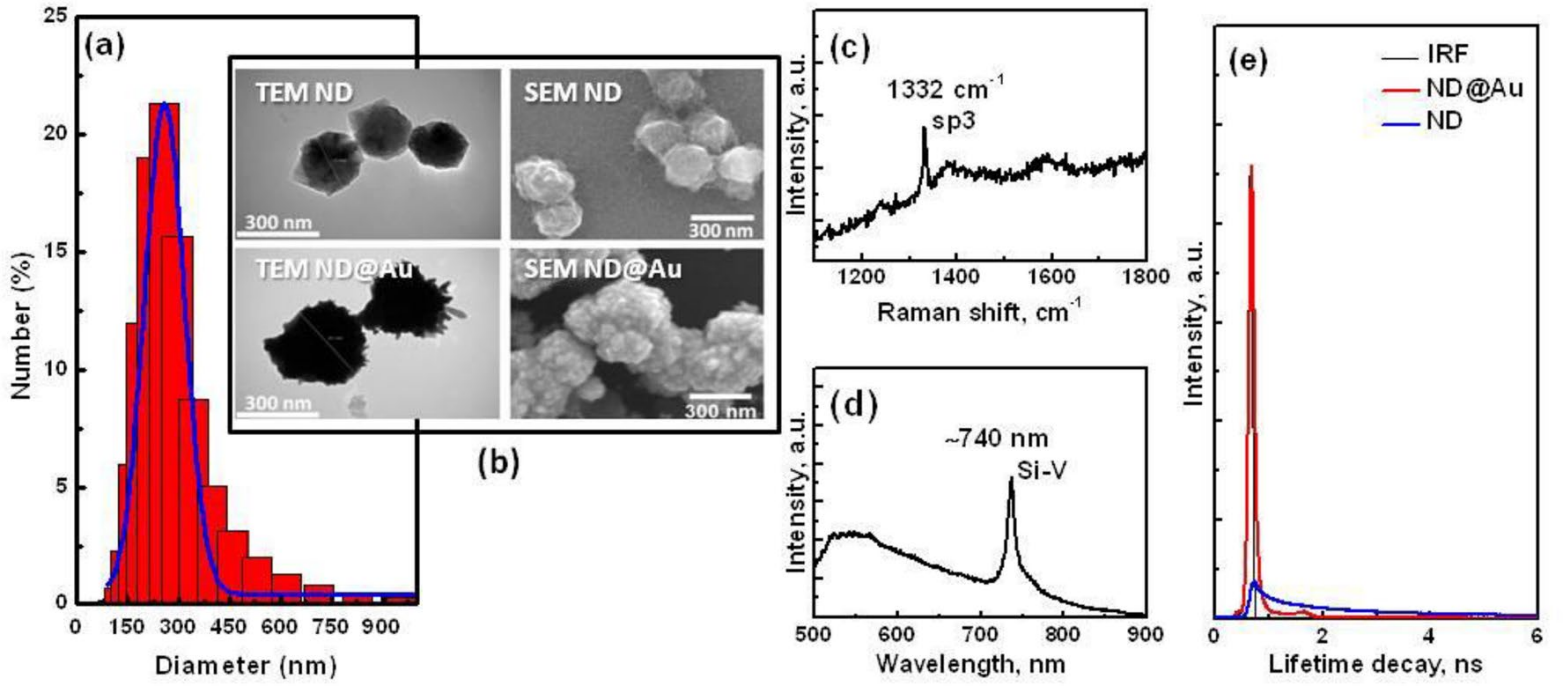
The Au@ND characterization: (**a**) The size distribution of ND@Au measured using DLS method, the average size is 282.8 ± 34.5 nm; (**b**) SEM and TEM images of ND and ND@Au. (**c**) Raman spectrum of ND@Au in characteristic ranges for ND Raman signals and (**d**) photoluminescence spectrum of ND@Au in the range containing ZPL of SiV PL; 488 nm wavelength laser excitation, laser power at the sample ~ 3 mW; (**e**) Fluorescence lifetime decays of ND and ND@Au at two-photon excitation, and calculated instrument response function (IRF), excitation 800 nm, laser power 1.5 mW for ND@Au, 14 mW for ND at the input to PicoHarb scanning system.

The average ND@Au particle size measured using dynamic light scattering (DLS) in distilled water is 282.8 ± 34.5 nm, the distribution is presented in Fig. 1a; and ζ-potential is negative and equals to – 29.5±0.3 mV at pH ~ 7. In the X-ray imaging (data not shown), the pixel resolution was 16 nm, FOV = 15 × 15 μm^2^, the averaged particle size is 0.25–0.35 μm, averaged from 24 particles; agree with the DLS results. Scanning and Transmission Electron microscopy images of the synthesized particles, Fig. 1b, are consistent with the size measurements. SEM and TEM images show the forming of the shell on the ND surface after Au deposition; TEM allows rough estimation of the shell thickness of the shown particles to be approximately 40 nm. The structure of the nanoparticles surface with irregularities and high local curvature due to the gold shell growth on the ND core is observed.

Figure 1c displays the Raman spectrum of ND in ND@Au with a dominant peak near 1332 cm^−1^, the phonon mode of sp^3^-hybridized carbon. The peak is narrow and isolated that characterizes well-ordered diamond structure of ND@Au diamond core. Figure 1d shows the photoluminescence spectrum of ND@Au NP with narrow and intense zero phonon line (ZPL) of SiV-center fluorescence in the near-infrared range, centered near 740 nm, far away from other ND fluorescence widen peaks. Both, ND fluorescence and Raman signals can be utilized for multimodal optical imaging with detection of one-photon excited signals. In the presented ND@Au, an additional imaging modality is provided by the Au shell. Luminescence at two-photon excitation from nanostructured gold was demonstrated and found highly efficient^19,20^. This was described as a sequential process of photons’ absorption and emission as result of the recombination of electrons in the sp-band and holes in the d-band^19^. Lifetimes of different gold nanostructures luminescence depend on shape and characteristic sizes of the gold nanostructure, on excitation power and were observed in the range from 0.25 to 2 ns^41,42^. Figure 1e depicts the fluorescence lifetime decay measured for ND@Au at 800 nm wavelength excitation and detection in the 450–650 nm range, estimated lifetime is about 0.1 ns. It is usually shorter than that of characteristic fluorescence lifetimes of biological systems’ autofluorescence and of most of fluorescence markers used for fluorescence imaging.

Figure 1e displays the fluorescence lifetime decays of ND and ND@Au. Lifetimes were estimated using 2-component model. For ND@Au the lifetime is estimated about 0.1 ns, comparable to the instrument response function (IRF). As such short lifetime was previously found characteristic for nanogold at two-photon excitation^43^, and Au emission is strong enough so we can suppose the observed lifetime of ND@Au is determined by Au shell. ND has two components of lifetime equal to 2.25 ns and 0.321 ns with close weights. Fluorescence lifetime comparable to the short component was observed for ND with low number of color defects and without enhanced fluorescence^44^. Emission of NV^0^ color center (ZPL 575 nm), the fluorescence of H^3^ (ZPL at 503 nm), H^4^ (ZPL at 496 nm) defects fall in the same interval, their fluorescence lifetimes were found longer^44^; and long lifetime component can be particularly from the defect fluorescence. However, the ND emission in this range at two-photon excitation is quite low in comparison with Au.

In the area near noble metal nanostructured surface, the incident electric field and the radiative decay rate of the fluorophore can enhance or quench due to surface plasmon resonance. The resulting effect depends on the distance between the nanostructured metal surface and fluorophore^45^. It has been shown that noble metal on surface of ND doesn’t affect fluorescence^46,47^, because color centers are embedded in the diamond lattice and are separated from the metal by non-diamond carbon surface layer which can rather create the condition for the fluorescence enhancement.

**Figure 2:**
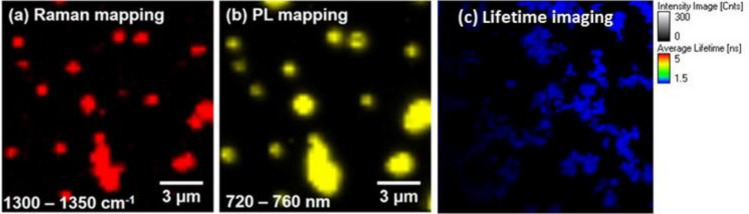
Imaging of ND@Au nanoparticles distributed along the substrate: mapping in pseudocolor of spatial distribution of (**a**) Raman signal in the 1300–1350 cm^−1^ range; (**b**) fluorescence in the 720–760 nm range. Excitation 488 nm wavelength laser, laser power at the sample ~ 3 mW; (**c**) Fluorescence lifetime imaging at 2-photon excitation with 800 nm wavelength laser, laser power at the sample ~ 0.5 mW.

The examples of imaging using ND@Au based on the spectroscopic properties of the components in ND@Au complex are demonstrated in Fig. 2. It displays the Raman images (Fig. 2a) and fluorescence (Fig. 2b) of ND core of ND@Au nanoparticles, using the same sample and set up; while 2-photon luminescence and FLIM is detected strongly predominantly from Au shell (Fig. 2c) in another set up/experiment/sample. This, for Raman and PL imaging ND spectroscopic properties are utilized while lifetime imaging capability of ND at the used conditions (two photon excitation and detection in the 450–650 nm range) is low. This kind of ND is poorly distinguishable with the biological object structures due to low emission intensity, and lifetime close to lifetime of endogenous fluorophores (which are predominantly in the range of 0.4–3 ns^48^). Below we consider ND@Au in applications as imaging agent using these optical facilities and also for hard X-ray full-field microscopy^49^ due to high contrast provided by Au shell. The optical properties of Au and synergetic properties of ND@Au complex allow considering more imaging modalities and methods of applications can be developed based on increased scattering signal of Au in area of plasmon resonance for microscopic imaging and Optical Coherence Tomography (OCT)^50^, using for photoacoustic imaging^10,28,33^, X-ray tomography and electron microscopy due to high contrast provided by Au shell, etc.^18^, as well as on the sensing abilities which are additionally increased by synergy between ND and Au^10^.

**Figure 3:**
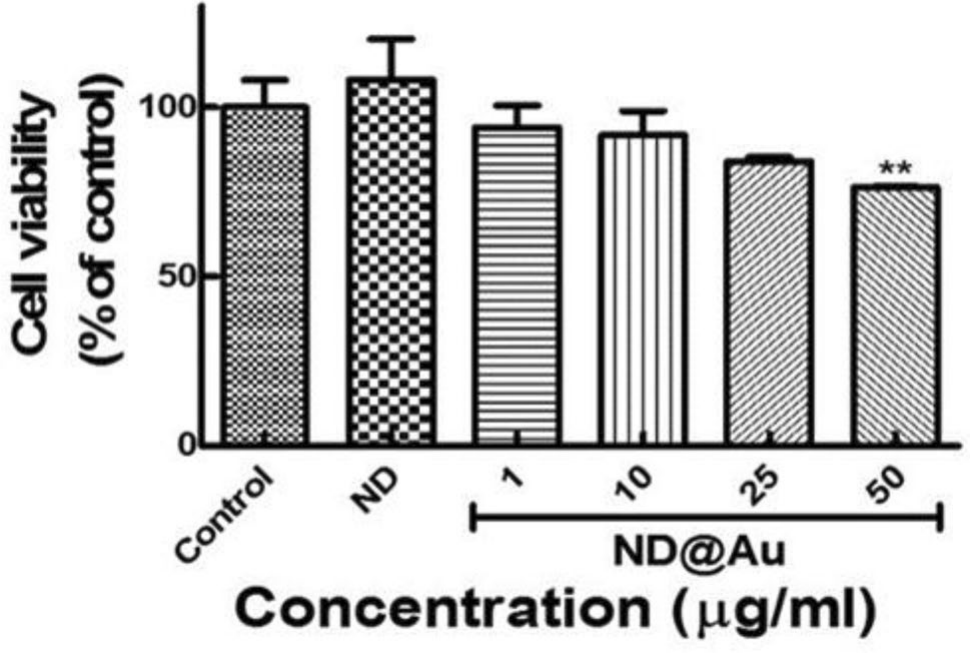
The ND@Au cytotoxicity estimated with MTT test. The cell viability was measured at A549 cancer cell incubation with various concentrations of ND@Au and ND for 24 h. Control is non-treated A549 cell culture. n = 6, (three experiments were repeated twice), ns, P > 0.05 was considered not significant, **P < 0.01. Data are mean ± SD.

Cytotoxicity of the ND@Au was analyzed using MTT assay with the viability of the A549 cell line (Fig. 3). The cell viability was measured after incubation of A549 cancer cell with various concentrations of ND@Au for 24 h. Negligible cytotoxicity was observed for ND and at ND@Au concentrations up to 10 µg/ml in the cell culture medium. With concentration increasing up to 50 µg/ml the observed cytotoxicity of ND@Au still is low.

Interaction of gold NP with cells, cells uptake and cellular responses, depend on the NP size, shape and surface properties (charge, coating, etc.)^51–53^. ND@Au particles have continuous gold shell^39^ and in terms of interaction with cells can be considered as gold spherical NP or nanoshells of quite large outer diameter, up to 200–300 nm and negatively charged.

Despite the fact that nanogold in general is often considered biocompatible, upon more thorough analysis of current data one can see that the problem of nanogold toxicity and biocompatibility is disputable. Contradictory results of investigations of Au nanoparticles toxicity reveals that large number of factors can affect Au nanostructures interaction with different kinds of biological systems, like the nanostructures type, size and shape of the particles, surface charge, surface topography and presence of surface defects, surface coating etc.^13–15^. Results of in-vitro analysis may differ with that obtained in-vivo. Thus, the conclusion about Au safety needs more concrete and systematic studies. However, among others, spherical nanoparticles and spherical nanoshells are shown to be the safest for cells. Such particles are quite biocompatible and safe at least in the absence of laser irradiation of specially selected wavelength, power, time exposure. Large spherical gold NP penetrate the cells via endocytosis and phagocytosis and are localized in endosomes in the cytoplasm on the cell periphery^14,16,52–54^. Thus, for the ND@Au particles used in this study we can suggest that they penetrate in cells, localize in endosomes in cytoplasm and create no damage or less damage than smaller particles can do, which directly interact with membrane and affect its structure^51–54^. All together allows us to conclude that ND@Au can be considered as a safe cellular marker.

### ND@Au visualization at interaction with biological systems

Observed Raman signal of sp^3^ bonded carbon of ND core, one-photon fluorescence of SiV with strong zero-phonon line near 740 nm, two-photon excited fluorescence of nanogold with short fluorescence lifetime and strong absorption of X-ray irradiation together with low cytotoxicity render Au@ND particles possible marker for bioimaging. Applications for Raman mapping, Fluorescence imaging, TP-FLIM and high-resolution hard-X-ray microscopy are demonstrated in cellular and developing zebrafish larvae (ZL) models.

**Figure 4:**
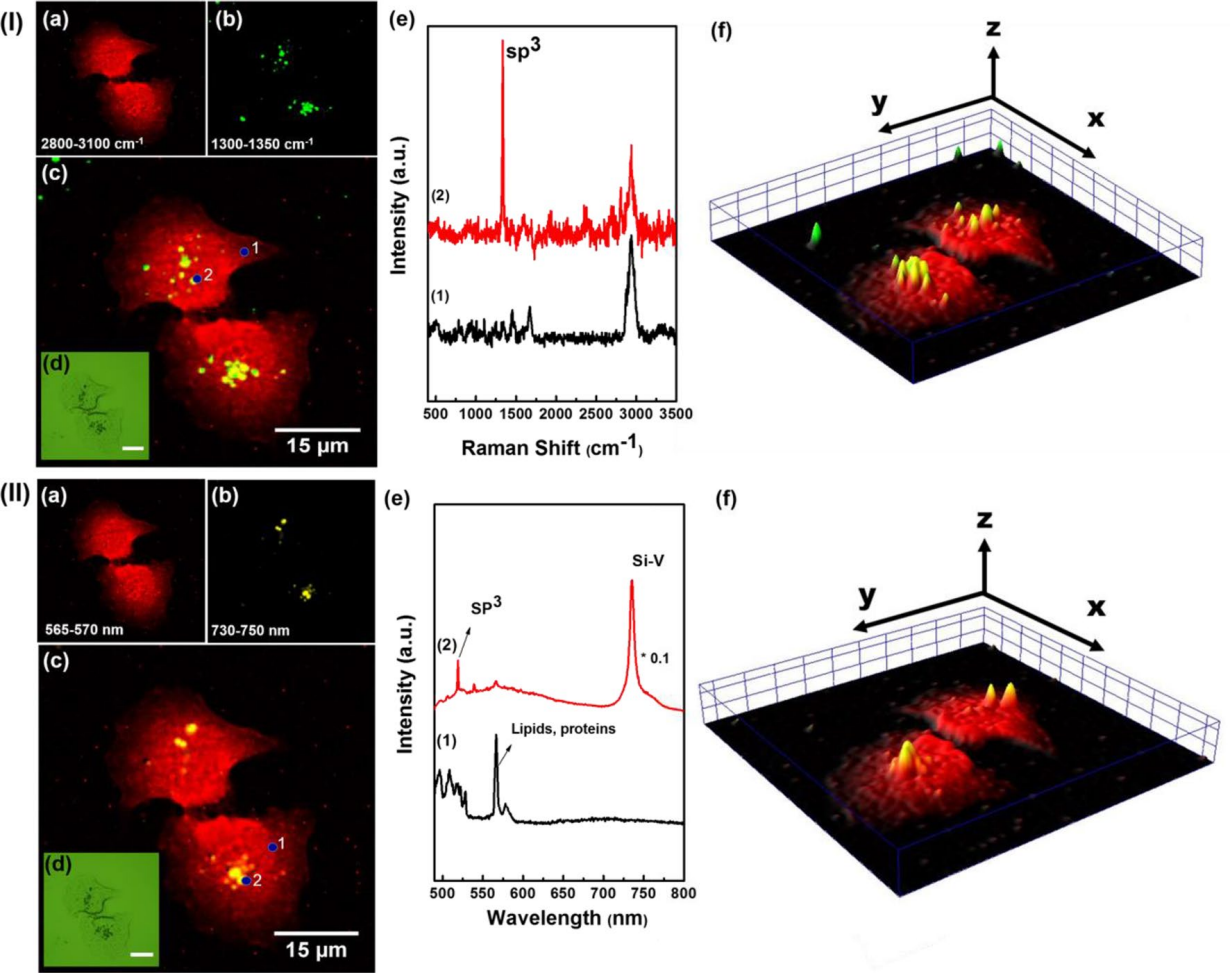
Visualization of ND@Au interaction with A549 cell via Raman mapping (**I**) and mapping of fluorescence (**II**). (**I**): (a) The cell is shown via mapping the distribution of intensity at 2800–3100 cm^−1^; (b) ND@Au NP are shown using ND Raman signal (mapping of the signal in range 1300–1350 cm^−1^); (c) merging of (a) and (b); (d) the optical image; (e) Raman spectra according to (c) measured in points marked 1 (cell) and 2 (the cell with ND@Au). (f) 3D Raman mapping reconstructed via the spectral signal intensity. (**II**) (a) The cell is shown via mapping the distribution of intensity at 565–575 nm; (b) ND@Au NP are shown via fluorescence of ND SiV center (730–750 nm); (c) merging of (a) and (b); (d) the optical image; (e) spectra measured from (c) point 1 (cell) and point 2 (the cell with ND@Au); (f) 3D mapping re-constructed via spectra intensity. The concentration of ND@Au was 20 µg/ml. excitation 488 nm.

**Figure 5:**
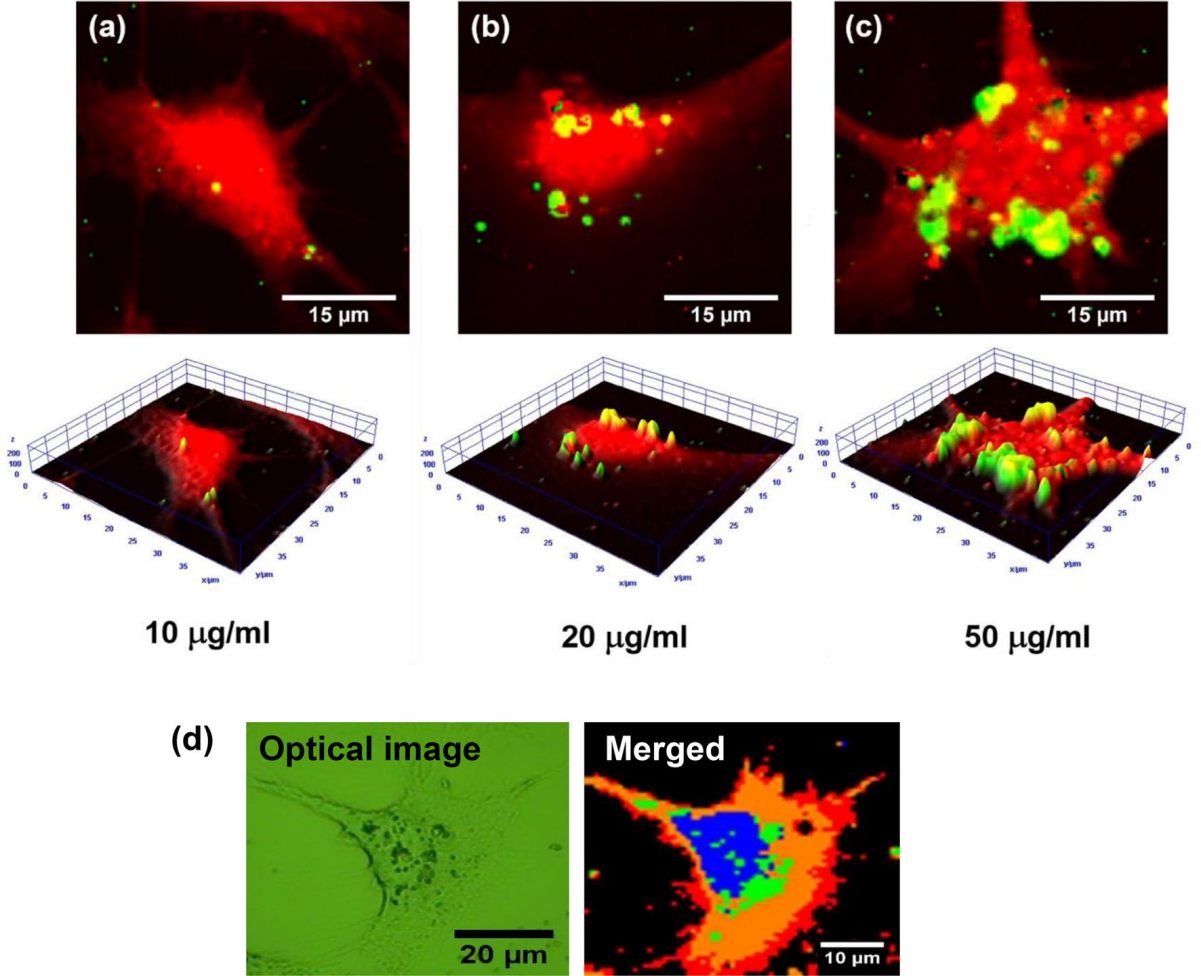
2D and 3D Raman mapping of A549 cell after incubation for 4 h with various concentrations of ND@Au: (**a**) 10 µg/ml, (**b**) 20 µg/ml and (**c**) 50 µg/ml. For cell imaging Raman intensity spatial distribution in the range 2800–3100 cm^−1^ is mapped (shown in red); ND@Au is shown via ND Raman signal in the 1300–1350 cm^−1^ range (green). (**d**) the reconstructed image of a Raman mapping from Principle Component Analysis (PCA) with K-means cluster analysis, shown the relative positions of each component, nucleus (blue), ND@Au (green), cytoplasm (red).

**Figure 6:**
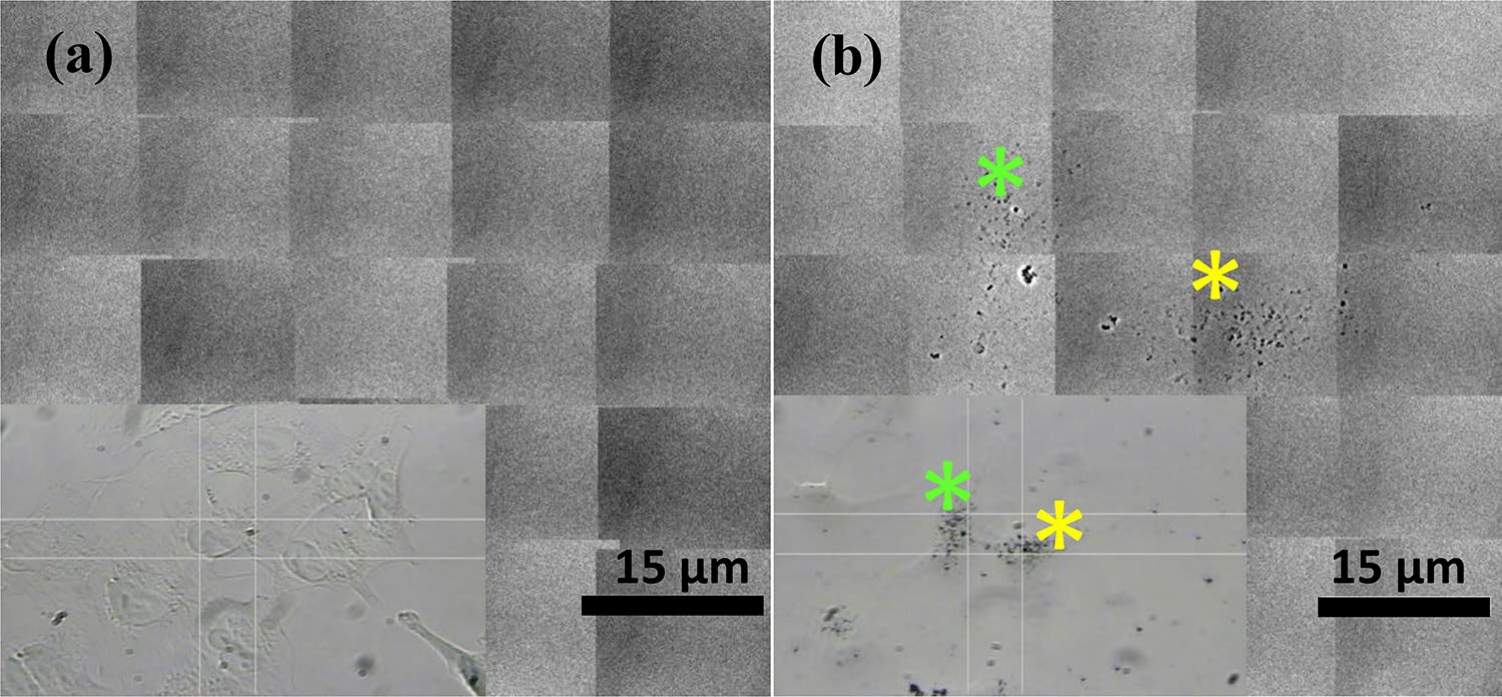
High resolution X-ray micrograph of A549 lung cancer cells cultured with ND@Au. Untreated control (**a**) and cells incubated with 50 µg/ml ND@Au for 24 h (**b**). The color stars mark the corresponding areas in X-ray and optical images.

**Figure 7:**
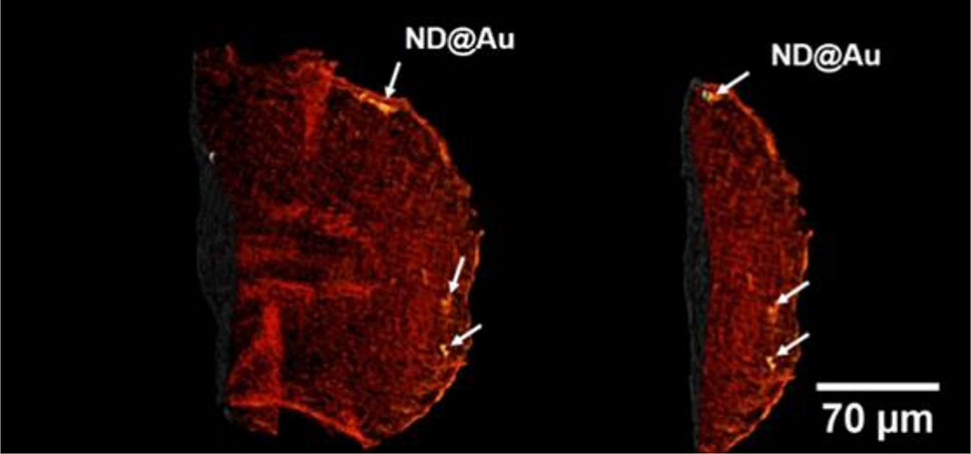
Tomographic volume rendered structure of hard X-ray micrographs of the 5-day-old zebrafish larvae’s tail. Injection of 2 mg/ml (4.6 nl) ND@Au into zebrafish embryo before 4 cell stage.

**Figure 8:**
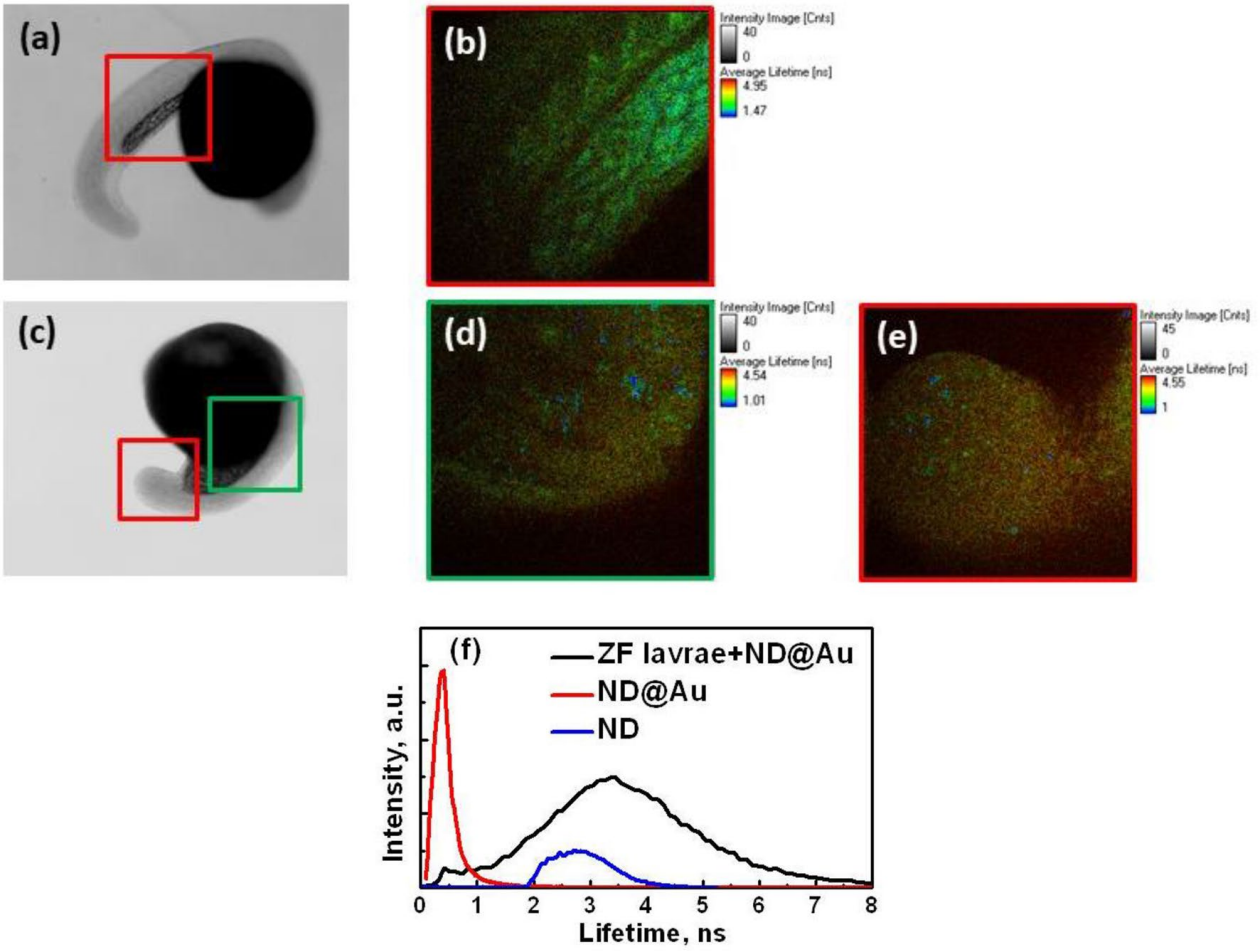
ND@Au imaging in the Zebra fish larvae (**a**) optical image and (**b**) FLIM of control larvae, without ND@Au; (**c**) optical image and (**d** green box; **e** red box) FLIM of larvae developing with injected ND@Au; (**f**) histograms of lifetime distribution along the image (**d**) (zebrafish (ZF) Larvae + ND@Au) and image (**c**) from Fig. 2 (ND@Au), and for control ND. Zebrafish larvae FLIM was performed at 800 nm wavelength laser excitation and laser power at the input to PicoHarb scanning system 20 mW.

Examples of using ND@Au NP for multimodal bioimaging are demonstrated in Figs. 4, 5, 6, 7 and 8. In Fig. 4, the mapping of spectroscopic signals of the A549 cells incubated with ND@Au is presented. For the cell visualization (Fig. 4Ia and IIa) one of characteristic Raman peak of biological cell is used at 488 nm wavelength excitation. This peak is attributed to C–H bonds in CH, CH_2_, CH_3_ groups of lipids and proteins. The Raman shift is observed in the 2820–2950 cm^−1^ range (Fig. 4Ie); or, in wavelength scale, at excitation 488 nm the same peak is observed at near 567–568 nm (Fig. 4IIe). To localize ND@Au in the cell both diamond Raman peak and fluorescence peak can be mapped. Figure 4Ib maps distribution on intensity of Raman peak from sp^3^ bonded carbon in ND (1300–1350 cm^−1^), while the Fig. 4IIb shows the mapping of fluorescence intensity of SiV ZPL (730–750 nm), depending on SiV concentration and relative positions in the particles. The merged images (Fig. 4Ic,IIc) show localization of the ND@Au in the cell and can be compared with the microscopic images in the inset (Fig. 4Id,IId). The spectra shown in Fig. 4Ie,IIe are measured in the points marked in the corresponding merged images and correspond predominantly (1) to cell, (2) to ND@Au NP. (Fig. 4If,IIf) are 3D mapping re-constructed via spectra intensity. From the imaging of the NP in the cell, using the spectral information, it is possible to analyze the spectra in every point (pixel) of the cell image. This analysis can be used for studying the cell state, as the Raman spectra contain data about the cellular components structure, composition, and functionality^55^.

Figure 5 illustrates the Raman mapping of cells with ND@Au at different concentrations of ND@Au. The presented images don’t provide direct information about NP localization inside the cell or on the membrane. One of the ways to confirm the ND@Au localization is confocal fluorescence imaging with ND detection via ZPL of SiV^26^. A z-scan Raman mapping also can reveal the location of the NP in cells^12^. In one of the Raman mapping, the reconstructed image (Fig. 5d) from Principle Component Analysis (PCA) with K-means cluster analysis, the relative positions of each component is more clear. In Fig. 5d, nucleus (blue), ND@Au (green), cytoplasm (red) can be seen at the same focal plane. In the Raman mapping, the focal plane is at the nucleus, so one can see the co-existence of nucleus and ND@Au, indicating ND@Au are inside the cytoplasm.

Au nanoparticles are considered as contrast-enhancing agent in X-ray imaging due to their high atomic number and excellent absorbing properties in corresponding range^56^. To demonstrate this, High-resolution X-ray imaging (TXM)^49^ was used to analyze the location of ND@Au in non-stained cellular and zebrafish larvae models. Figure 6 shows the optical images (insets) and the high-resolution X-ray micrographs which were obtained by scan along A549 lung cancer cell (Fig. 6a) and the cell incubated with 50 µg/ml ND@Au for 24 h (Fig. 6b). Differently to the non-stained cell, ND@Au NP are well-detectable in X-ray image and are co-localized with the cell in optical image. ND@Au are well suitable for TXM studies without further staining. The use of a coherent (synchrotron) source of X-rays, with the magnification provided by high-resolution Fresnel zone-plate objectives can produce high-quality microradiographs of human and mouse cells with 29 nm Rayleigh spatial resolution and the tomographic reconstruction could be implemented with a final resolution level suitable for subcellular features^49^.

The zebrafish (Danio rerio) is a widely used vertebrate model for toxicity studies. We visualize ND@Au interaction with developing zebrafish embryo (after fixation) using the high-resolution X-ray imaging and FLIM at 2-photon excitation. Figure 7 shows tomographic volume rendered structure of hard X-ray micrographs of the 5-day-old zebrafish larvae’s tail. After the injection of 2 mg/ml (4.6 nl) ND@Au into zebrafish embryo in early stage of development (before 4 cell stage) nanoparticles are distributed predominantly in the developed blood vessels, where they can be carried with blood stream along the body^57^.

Figure 8 presents ND@Au visualization in the zebrafish larvae (48 hpf) developing after ND@Au injection before 4 cell stage via FLIM at two-photon excitation. Figure 8a,b show microscopic image and FLIM for control larvae without the NP. Microscopic image and FLIM of larvae with injected ND@Au is shown in Fig. 8c–e. ND@Au are clearly observed due to short fluorescence lifetime of nanogold. The emission from Au of ND@Au is intense and lifetime significantly differs with the larvae autofluorescence, so the NP are well detected in this organism. The images show ND@Au distribution in the zebrafish larvae body. The presence of short-lifetime NP also histogram of distribution of lifetimes along images reveals, demonstrating the peak, corresponding to ND@Au (Fig. 8f). The histogram for the zebrafish larvae (ZL + ND@Au) corresponds to image in Fig. 8d, showing two peaks. One of them corresponds to very short lifetimes in range 0.1–0.2 ns. The second one is wide and centered near 3.4 ns, it should be attributed to the larvae autofluorescence. The histogram for ND@Au (Fig. 8e) corresponds to fluorescence lifetime data shown in Fig. 2c and confirms that the small peak in the zebrafish larvae + ND@Au histogram in the short lifetimes range is revealing the presence of ND@Au. The histograms show numerical data for the lifetimes, available for quantitative analysis, and illustrate once more the separation between ND@Au and the organism signal and the ND@Au clear detection in contrast to ND particles with lifetime comparable with the biological object fluorescence.

Effective concentration of ND@Au just after injection of 4.6 nl in concentration of 2 mg/ml in early-stage zebrafish embryo is about 18 µg/ml and multiple times decreases with the embryo growth. Although this initial concentration was shown non-cytotoxic using MTT test, the microscopic image for the larvae with ND@Au, shown in the Fig. 8c, reveals some morphological disorder (malformation during embryonic development in the presence of ND@Au). Thus, observed effect of ND@Au on developing zebrafish embryo indicates that the development of ND@Au for bioapplications requires, among other things, further study of ND@Au effects on living systems at different levels of developments.

The results show possibilities to reveal the NP localization both in cellular model and in multicellular developing object and to use ND@Au as a high contrast imaging agent. Additionally, one of the advantages of FLIM is that the lifetimes distribution and their changes allow monitoring and quantification of metabolic state of the living cell based on endogenous fluorophores’ lifetimes distribution^47^ simultaneously with analysis of interaction with NP. Another potential advantage is multifunctional (theranostic) applications, as the combination of nanogold and laser treatments is used for development of methods photothermal and photodynamic therapy. Optical and plasmonic properties of nanogold, being important both for bioimaging/biosensing and for photo treatment, highly depend on the NP geometry^54^. Thus, surface plasmon resonance of gold nanoshells in the core–shell Au–Si structure with core size about 100 nm was shown to be controlled by varying the shell thickness. In this case, decreasing the gold shell thickness resulted in surface plasmon resonance red shifted, which was attributed to the increased coupling between the inner and outer shell surface plasmons for thinner shell particles^52^. The surface plasmon resonance frequency found depends on the ratio of the shell-to-core thickness^53^. While decreasing the gold shell thickness at a constant outer diameter of the shell results in surface plasmon resonance red shifted, for hollow gold nanoshells an increase in the shell thickness at a constant outer diameter causes a blue shift in plasmon resonance peak^58^. Thus, optical and plasmonic properties of the core–shell ND@Au particles can be tuned for optimally successful multifunctional applications.

In conclusion, ND@Au hybrid core–shell nanoparticles are synthesized and characterized in terms of their multifunctional bioapplications. Different imaging modalities based on the combination of optical and spectroscopic properties of the hybrid nanosystem are demonstrated in cellular and developing zebrafish larvae models. ND@Au nanoparticles are shown as convenient imaging agent for Raman mapping via sp^3^ carbon Raman signal, one-photon Fluorescence imaging via fluorescence of SiV color centers in the nanodiamond core, two-photon Fluorescence imaging and FLIM and high-resolution X-ray microscopy utilizing the plasmonic properties of Au nanoshell. This complex can have potential for applications both in the cellular (Raman, 2P-FLIM, confocal, etc.) and in animal (X-ray) models. The properties of ND@Au allow discussion of combining of the imaging facilities with other theranostic functionalities.

## Materials and methods

### Synthesis of SiV-doped nanodiamond

SiV-doped nanodiamonds were synthesized using plasma enhanced chemical vapor deposition (PECVD) technique with a conventional H_2_/CH_4_ mixture of typically 1% CH_4_ to 99% H_2_. The applied power was 1 kW, pressure 12 mbar and the substrate temperature was adjusted to 400 °C to prevent carbide formation. A 3-inch Si-wafer was used as doping source for Si. The growth rate was about 12 nm/h. The nanoparticle produced had a typical diameter of around 200 nm. These particles were removed from the wafer and dispersed in water solution to form a stable colloid after air oxidation at 480 °C.

### ND@Au synthesis and characterization

ND@Au were synthesized by Immagina Biotechnology srl using a proprietary procedure. Briefly, 0.1 mg of the (SiV) nanodiamonds were dispersed in 10 ml water solution. Then 800 µl of 10 mM HAuCl_4_ water solution were introduced and the mixture was incubated for 2 min under stirring at room temperature. The reducing agent hydroxylamine hydrochloride NH_2_OH*HCl (500 µl, 500 mM) was then added to the suspension. To start the gold reduction, the pH of the suspension was increased by addition of NaOH solution (pH 12). After 5 min, the solution color changed from white to pale red. The suspension pH was then increased to 10 with NaOH solution at pH 12. Drop wise addition of 10 mM HAuCl_4_ water solution in the mixture under stirring at room temperature cause the change of the color from pale red to grey-blue. The suspension was purified by 10 min centrifugation at 12,000 rpm and washed three times with distilled water.

The particles prepared were characterized in terms of their use for multimodal bio-imaging. The particle size and ζ-potential were analyzed using dynamic light scattering method, with the Zetasizer Nano ZS (Malvern Instruments, Malvern, UK) equipped with 633 nm wavelength He–Ne laser (4 mW) and detection angle 173°, in suspensions in distilled water. The pH of the suspensions was kept at 7 and measured with a SENTRON pH meter (Titan, Taiwan).

Scanning Electron Microscopy (SEM, Hitachi S3400, Japan) and Transmission electron microscopy (TEM, JEM-1400 JEOL, Japan) were employed to analyze the surface morphology and particle structure of the particles. The spectroscopic properties and structure of ND@Au were analyzed from the fluorescence and Raman spectra measured and Raman mapping performed with a confocal Raman spectrometer α-SNOM (WITec, Germany) with a diode pump solid state (DPSS), 488 nm wavelength laser excitation. Fluorescence lifetime analysis was performed using a system and method previously described^44,57^. In short, a tunable Ti–sapphire laser (Chameleon Ultra, Coherent, USA) was used for 2-photon luminescence excitation with 800 nm wavelength; pulse duration 140 fs; repetition rate 80 MHz. A two-dimensional scanner (EINST Technology) was used for imaging. The excited signal was collected in 450–640 nm range with a single-photon counting system (PicoHarp 300, PicoQuant Germany) and Photomultiplier Detector Assembly PMA-C-192-M, and an objective UPlanFLN 40×/0.75 (Olympus, Japan) was used. Fluorescence lifetime of ND@Au and instrument response function (IRF) were estimated using SymphoTime software (PicoQuant Germany).

For spectroscopic and SEM measurements, ND@Au water suspension of concentration in the range 0.1–1 mg/ml was dropped on Si substrate, or for FLIM on cover glass and dried.

### A549 cell culturing and preparation for Raman spectroscopy and X-ray imaging

Human lung alveolar carcinoma epithelial cell A549 was obtained from Biore-source Collection and Research Center (BCRC) in Taiwan. A549 cells were cultured in RPMI1640 medium (Gibco, Invitrogen, UK). The medium was supplemented with 2 mM l-glutamine (Invitrogen, USA), 1.5 g/L sodium bicarbonate (Sigma, UK), 10% fetal bovine serum (Gibco/Life Technologies, Carlsbad, CA, USA). Cells were maintained under standard cell culture conditions in an incubator (Galaxy 170S, Eppendorf, USA) containing 95% air and 5% CO_2_ at 37 °C humid environment. Culture medium was re-placed with fresh medium every 48 or 72 h. Cells were detached by treatment with 0.5% trypsin and 2.6 mM ethylenediaminetetraacetic acid (EDTA) (Gibco/Life Technologies, Carlsbad, CA, USA); cultures were subcultured routinely at approximately 80% confluence.

The A549 cells were treated with ND@Au, each sample was added to the medium at concentration of 10, 20 and 50 µg/ml. Cells were incubated together with the NP samples for 2–24 h. Unreacted samples were removed by washing. The cells with ND@Au adhered on the coverslips were used for microscopic investigations.

For Raman mapping and X-ray imaging, the ND@Au treated A549 cells adhered on the coverslips were fixed by 4% paraformaldehyde with following sequential dehydrated by 30%, 50%, 70%, 95% and 100% gradient ethanol before mapping or imaging.

### Cell cytotoxicity test

The cytotoxicity was estimated by MTT assay using ND or ND@Au and A549 cell line. A549 cells were seeded in quantity of 4 × 10^5^ cells/well in a 96-well microtiter plate in RPMI1640 medium (Gibco, Invitrogen, UK) 2 mM l-glutamine (Invitrogen, USA), 1.5 g/l sodium bicarbonate (Sigma, UK), 10% fetal bovine serum (Gibco/Life Technologies, Carlsbad, CA, USA) and incubated at 37 °C and 5% CO_2_ for 24 h. After incubation, the medium was removed. Then the cells were treated with a fresh medium containing 50 µg/ml of ND or different ND@Au concentrations (1–50 μg/ml) for 24 h. After this procedure, the medium was removed and the cells were treated for 4 h with MTT reagent (with the concentration of 2.5 mg/ml). The surviving cells converted MTT-agent to formazan, a blue-purple color when dissolved in dimethyl sulfoxide. The solution was removed and 200 μl of dimethyl sulfoxide (DMSO) was added to dissolve the formazan. The optical absorption of the treated cells and control (non-treated) cells was measured at 570 nm (O.D. 570) with ELISA reader (MRX revelation Microplate Reader, DYNEX, USA). Relative percentages of surviving cells were calculated by the dividing the absorbance of the treated cells with that of the control measured in each experiment.

### Zebrafish maintenance, injection of ND@Au into zebrafish embryo and imaging

Mature zebrafish were raised at the zebrafish facility of Tze Chi University, Hualien, Taiwan. All animal experiments in this study were approved by Institutional Animal Care and Use Committee of Tze Chi University and performed in accordance with the “Animal Research: Reporting in vivo Experiments” guideline issued by animal ethic committee. All methods were carried out in accordance with relevant guidelines and regulations.

Zebrafish (AB strain) adults were maintained at 28 °C in aerated aquarium with a photoperiod of 14 h light and 10 h dark. In each mating setup two males and four females were used. Embryos were collected within 20 min after mating. Embryos were cleaned by fresh-water and collected for ND@Au microinjections. The samples were loaded into zebrafish embryos by microinjection under a dissecting microscope (SMZ745T, Nikon, Japan). The 2 mg/ml ND@Au water solution was injected into the animal pole region of zebrafish embryos with microinjector (NANOJECT II, Drummond Scientific Company, Philadelphia, USA) before 4-cell stage (1 h post fertilization, hpf). The microinjection pipette was calibrated every time before microinjection. The injected volume was approximately 4.6 nl. After microinjection, the embryos were transferred to the 10 cm culture dish along with 20 ml of system water (embryo culture water) and incubated at 28 °C under dark conditions for further development.

For in-vivo observation with FLIM of the ND distribution inside the zebrafish larvae, the samples were prepared as described previously^57^. After the microinjection of ND@Au the zebrafish larvae was placed into 3 ml embryo culture solution in the glass culture dishes added with 0.5 ml of Tricaine methanesulfonate (125 μg/ml). The zebrafish embryo imaging can be obtained in-vivo until the larvae start to move. After that the samples has to be fixed.

To prepare zebrafish larvae for FLIM and X-ray tomographic imaging, 5-day-old zebrafish larvae samples were stained using the Golgi method. In brief, the sample was fixed with 4% paraformaldehyde overnight and then immersed in 2.5% potassium dichromate in the dark. After three days, the samples were transferred into 0.1 M silver nitrate for 3 additional days in the dark. After sequential dehydration by 30%, 50%, 70%,95%, 100% gradient ethanol and acetone, the specimens were embedded in an epoxy resin (Sigma) according to the specimen preparation described previously^44^.

### Optical measurements of ND@Au interaction with cell and zebrafish embryo

The interaction of ND@Au with the cell was visualized via mapping of the spatial distribution of the nanodiamond spectroscopic signal measured using a Raman spectrometer (α-SNOM, WITec, Germany). The intensity of Raman spectrum in the 1300–1350 cm^−1^ region (the region of phonon mode of sp^3^ bonded carbon at 1332 cm^−1^) or fluorescence intensity in the 720–760 nm range (SiV, ZPL at 739 nm) were used for the mapping. The cells treated or non-treated (control) with ND@Au were prepared for the imaging measurements as described above; the cells were visualized via Raman mapping of cell signal in 2800–3000 cm^−1^ region of the C–H, C–H_2_ groups vibrations of lipids and proteins.

To obtain high-resolution and high contrast images of the A549 cell and zebrafish larvae interacting with ND@Au, the phase contrast radiology method was employed, details described elsewhere^49^. In short, the beamline TLS01B in the National Synchrotron Radiation Research Center (NSRRC, Hsinchu, Taiwan) was used, equipped with ultrahigh resolution transmission X-ray microscopes (TXM). The X-ray source was a 5 T superconducting wavelength shifter (SWLS) inserted along the 1.5 GeV storage ring at NSRRC ring. X-rays in TXM generated was focused on the specimen, the transmitted X-rays were detected by a CdWO_4_ single-crystal scintillator and converted to visible images. These images are then magnified by an optical lens captured and stored by a CCD camera. The total magnification in TLS01A reached 900–2400×. The resulting single image (typically 1600 × 1200 pixel, horizontal field of view = 500 μm) taking for biological samples with low X-ray absorption was 500 ms or less.

Fluorescence lifetime imaging (FLIM) of the zebrafish embryo developed after injection of ND@Au was acquired using set-up described in the “ND@Au visualization at interaction with biological systems”, and details described previously for zebrafish embryo studies^57^. Objective Plan Apo 20×/0.70(Olympus, Japan) was used in this measurement. Fluorescence lifetime images of zebrafish embryo were obtained and analyzed using SymphoTime software.
